# Bamboo Biochar and Sodium Silicate Alleviate Oxybenzone-Induced Phytotoxicity via Distinct Mechanisms for Sustainable Plant Protection

**DOI:** 10.3390/plants14152382

**Published:** 2025-08-02

**Authors:** Chuantong Cui, Wenhai Yang, Weiru Dang, Ruiya Chen, Pedro García-Caparrós, Guoqun Yang, Jianhua Huang, Li-Jun Huang

**Affiliations:** 1 Key Laboratory of Forest Bio-Resources and Integrated Pest Management for Higher Education in Hunan Province, Key Laboratory of Cultivation and Protection for Non-Wood Forest Trees, State Key Laboratory of Utilization of Woody Oil Resource, College of Forestry, Central South University of Forestry and Technology, Changsha 410004, China; 2 Hunan Academy of Forestry, Changsha 410018, China; 3 Department of Forestry, Bangor College, Changsha 410004, China; 4 Higher Polytechnic School, University of Almeria, 04120 Almeria, Spain

**Keywords:** ultraviolet filter, oxybenzone, biochar, sodium silicate, oxidative stress

## Abstract

Oxybenzone (OBZ), an organic ultraviolet filter, is an emerging contaminant posing severe threats to ecosystem health. Using tobacco (*Nicotiana tabacum*) as a model plant, this study investigated the alleviation mechanisms of exogenous silicon (Na_2_SiO_3_, Si) and bamboo-based biochar (Bc) under OBZ stress. We systematically analyzed physiological and biochemical responses, including phenotypic parameters, reactive oxygen species metabolism, photosynthetic function, chlorophyll synthesis, and endogenous hormone levels. Results reveal that OBZ significantly inhibited tobacco growth and triggered a reactive oxygen species (ROS) burst. Additionally, OBZ disrupted antioxidant enzyme activities and hormonal balance. Exogenous Bc mitigated OBZ toxicity by adsorbing OBZ, directly scavenging ROS, and restoring the ascorbate-glutathione (AsA-GSH) cycle, thereby enhancing photosynthetic efficiency, while Si alleviated stress via cell wall silicification, preferential regulation of root development and hormonal signaling, and repair of chlorophyll biosynthesis precursor metabolism and PSII function. The mechanisms of the two stress mitigators were complementary, Bc primarily relied on physical adsorption and ROS scavenging, whereas Si emphasized metabolic regulation and structural reinforcement. These findings provide practical strategies for simultaneously mitigating organic UV filter pollution and enhancing plant resilience in contaminated soils.

## 1. Introduction

Organic ultraviolet (UV) filters are widely used in modern sunscreen products, plastics, and personal care products. With the extensive use of these products, organic UV filters are frequently detected in surface water, sewage, and activated sludge [1,2,3]. These substances are highly lipophilic and also possess endocrine-disrupting effects and toxicological impacts [4,5]. As a result, UV filters are recognized as emerging contaminants [6]. With their continuous and substantial use and discharge, organic UV filters gradually infiltrate various aquatic environments and soil, posing a significant threat to the entire ecosystem [7]. Oxybenzone (OBZ), as the most widely used organic UV filter, plays a crucial role in absorbing UV rays and preventing skin sunburn. However, its negative effects on the ecological environment are becoming increasingly prominent. In areas with severe pollution, the detected concentration of OBZ in water bodies is as high as 3.316 mg/L, while in soil, it reaches 35 mg/kg [8]. Studies have shown that OBZ exhibits multiple biological toxicities, particularly in its toxic effects on aquatic and terrestrial organisms. In aquatic ecosystems, OBZ can adversely affect the growth and reproduction of fish, crustaceans, and algae, thereby disrupting the aquatic ecological balance [8,9,10,11]. In terrestrial ecosystems, OBZ can cause numerous detrimental effects on the growth and development of plants. Specifically, OBZ can inhibit photosynthesis in plants, impairing the photosynthetic energy conversion process [12]. Additionally, it can interfere with the hormonal balance within plants, leading to stunted growth, yellowing of leaves, reduced biomass, and severe damage to the antioxidant system [13]. Therefore, actively exploring strategies for plants to cope with OBZ stress is of paramount importance for effectively protecting the ecological environment and maintaining ecological balance. Plants are highly sensitive to various environmental stresses during their growth and development.

Silicon (Si) is one of the abundant elements in the Earth’s crust and is widely distributed in soil in the form of SiO_2_ in nature [14]. Although Si is not an essential nutrient element for plant growth and development, numerous research results show that it has a positive regulatory effect on plant growth and development, especially in alleviating various biotic and abiotic stresses, such as salt stress [15], drought stress [16], freezing stress [17], nutrient deficiency stress [18], heavy metal stress [19], pest and pathogen stress [20], etc. Its specific mechanism of action is mainly manifested in that exogenous silicon can effectively enhance stress resistance by improving plant growth, photosynthesis, and antioxidant metabolism [21].

Additionally, exogenous silicon can enhance the structural stability of plant cell walls. It can bind with pectin and cellulose in the plant cell walls, forming a more robust cell wall structure [22]. This enhanced cell wall structure can resist mechanical damage and pathogen invasion, thereby improving the plant’s disease and pest resistance.

Biochar (Bc) is a porous carbonaceous material produced by the pyrolysis of biomass under high-temperature, oxygen-free conditions, and is highly regarded as a soil amendment [23]. It possesses properties such as abundant pores, high specific surface area, high ash content, alkalinity, and strong stability, among other physical characteristics [24]. In recent years, the application of Bc in environmental remediation and agricultural production has become increasingly widespread [25]. Research indicates that Bc can significantly improve soil structure and physicochemical properties, enhancing the soil’s water retention capacity and fertility levels [25]. Simultaneously, Bc can also adsorb harmful substances in the soil, reducing their toxic effects on plants [26]. This adsorption capability can mitigate the toxic effects of harmful substances on plants, protecting their growth and development. Furthermore, biochar demonstrated the ability to alleviate stress conditions by improving plant growth, photosynthesis, and antioxidant metabolism, as well as enhancing the absorption and utilization of mineral nutrients, which partially overlaps mechanistically with exogenous silicon and other common stress mitigators [27].

Based on the mitigation effects of these two stress alleviators on both biotic and abiotic stresses, they bring significant insights to this study. In a previous study, we explored the toxic mechanisms of organic UV filters from multiple dimensions. The results indicate that OBZ could cause harm to plants by interfering with photosynthetic efficiency, inducing oxidative stress responses, and disrupting hormone balance [28]. Building upon this research, we employed exogenous Si and bamboo-based Bc as stress alleviators to investigate their mitigating effects on plants subjected to stress from the organic UV filter OBZ. The results indicate that exogenous Si and Bc exhibit substantial potential as mitigators for tobacco plants under stress from the organic UV filter OBZ, and they share a high degree of similarity in their physiological and biochemical mechanisms. Therefore, the findings of this study can provide coping strategies for plants encountering the stress of the organic UV filter OBZ and offer new strategies for environmental protection and ecological restoration.

## 2. Results

### 2.1. Phenotypic Parameters

**Figure 1:**
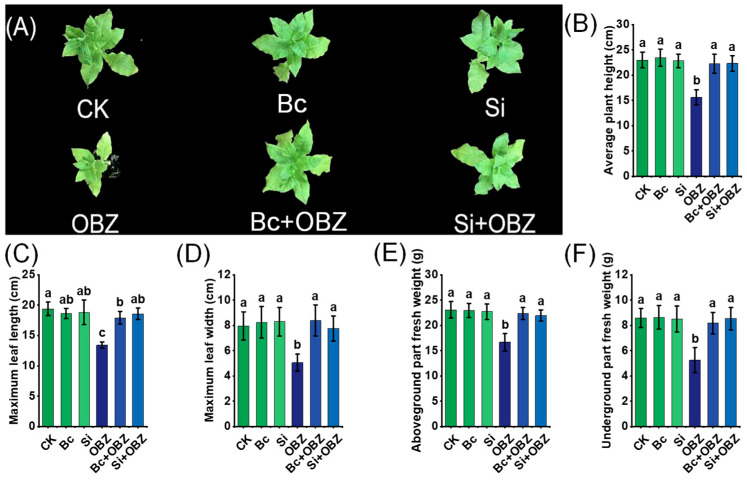
Effects of bamboo biochar (Bc) and sodium silicate (Si) on the growth of tobacco under oxybenzone (OBZ) stress. (**A**) Representative phenotypes of tobacco seedlings under different treatments. (**B**–**F**) Quantitative analysis of growth parameters including (**B**) average plant height, (**C**) maximum leaf length, (**D**) maximum leaf width, (**E**) aboveground fresh weight, and (**F**) underground fresh weight. Data are presented as means ± standard deviation (SD, n = 3). Different lowercase letters indicate significant differences among treatments (*p* < 0.05) according to one-way ANOVA followed by Duncan’s test.

In this study, OBZ treatment exhibited significant inhibitory effects on tobacco growth. Compared to the control group (CK), OBZ-treated plants displayed marked morphological abnormalities, including significantly reduced leaf size (*p* < 0.05) and chlorosis (yellowing due to chlorophyll degradation). Plant height, maximum leaf length, maximum leaf width, aboveground biomass, and underground biomass decreased by 32.08% (*p* < 0.05), 30.74% (*p* < 0.05), 36.49% (*p* < 0.05), 27.83% (*p* < 0.05), and 38.76% (*p* < 0.05), respectively, indicating that OBZ markedly suppressed plant growth by disrupting cell division, photosynthesis, and nutrient allocation (Figure 1).

Exogenous application of bamboo-based Bc and sodium silicate (Na_2_SiO_3_) effectively alleviated OBZ-induced growth inhibition. In Bc + OBZ-treated plants, plant height, maximum leaf length, maximum leaf width, aboveground biomass, and underground biomass recovered by 42.68% (*p* < 0.05), 33.52% (*p* < 0.05), 65.88% (*p* < 0.05), 34.29% (*p* < 0.05), and 55.69% (*p* < 0.05), respectively, compared to the OBZ group. Notably, maximum leaf width and underground biomass exhibited the most pronounced recovery (*p* < 0.05), suggesting that Bc likely mitigates OBZ toxicity by adsorbing toxic molecules or improving rhizosphere microenvironments to enhance nutrient uptake. In the Si + OBZ group, the corresponding parameters increased by 42.89% (*p* < 0.05), 38.33% (*p* < 0.05), 53.36% (*p* < 0.05), 31.69% (*p* < 0.05), and 62.55% (*p* < 0.05), respectively. Sodium silicate likely alleviated stress by enhancing cell wall silicification or regulating ion transport to optimize root system development.

### 2.2. Effects on Reactive Oxygen Species (ROS) Levels

**Figure 2:**
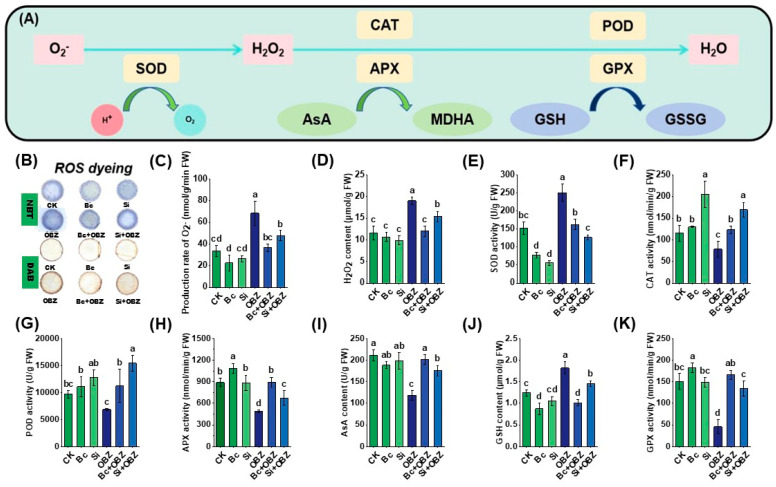
Effects of bamboo biochar (Bc) and sodium silicate (Si) on reactive oxygen species (ROS) accumulation and antioxidant enzyme activities in tobacco under oxybenzone (OBZ) stress. (**A**) Proposed model of the antioxidant defense system, including the enzymatic and non-enzymatic pathways for ROS scavenging. (**B**) Histochemical detection of ROS using DAB and NBT staining in leaves under different treatments (visually discernible differences). (**C**) The generation rate of superoxide anion (O_2_^−^). (**D**) The content of hydrogen peroxide (H_2_O_2_). (**E**) Superoxide dismutase (SOD) activity. (**F**) Catalase (CAT) activity. (**G**) Peroxidase (POD) activity. (**H**) Ascorbate peroxidase (APX) activity. (**I**) The content of ascorbic acid (AsA) content. (**J**) The content of reduced glutathione (GSH). (**K**) Glutathione peroxidase (GPX) activity. Data are presented as means ± standard deviation (SD, n = 3). Different lowercase letters indicate significant differences among treatments (*p* < 0.05) according to one-way ANOVA followed by Duncan’s test.

ROS generation rate assays and histochemical staining analyses (Figure 2) revealed that OBZ treatment significantly exacerbated oxidative stress in tobacco leaves. Compared to the untreated group (CK), the superoxide anion (O_2_^−^) production rate and hydrogen peroxide (H_2_O_2_) concentration in the OBZ group increased by 104.25% (*p* < 0.05) and 64.87% (*p* < 0.05), respectively, indicating that OBZ induces ROS burst by disrupting mitochondrial electron transport chains or activating NADPH oxidases. Histochemical staining further confirmed excessive ROS accumulation, with OBZ-treated leaves exhibiting intensified O_2_^−^-specific fluorescence signals and expanded H_2_O_2_-positive areas (DAB staining).

Exogenous Bc markedly suppressed ROS generation. O_2_^−^ production rate and H_2_O_2_ concentration decreased by 46.53% (*p* < 0.05) and 36.64% (*p* < 0.05), respectively, compared to the OBZ group. In contrast, Na_2_SiO_3_ showed weaker mitigation, reducing O_2_^−^ and H_2_O_2_ by 30.15% (*p* < 0.05) and 18.99% (*p* < 0.05), respectively. This divergence likely stems from distinct mechanisms: Bc may adsorb OBZ or directly scavenge free radicals to disrupt ROS chain reactions, whereas Si likely alleviates oxidative damage indirectly by enhancing cell wall mechanical barriers or activating antioxidant enzymes such as superoxide dismutase (SOD) and catalase (CAT).

### 2.3. Antioxidant System

Analysis of key antioxidant system indicators (Figure 2) demonstrated that OBZ treatment significantly altered antioxidant enzyme activities and redox balance. Compared to the control (CK), SOD activity and glutathione (GSH) content in the OBZ group increased by 64.65% (*p* < 0.05) and 46.57% (*p* < 0.05), respectively, indicating that OBZ-induced oxidative stress activated SOD-mediated O_2_^−^ scavenging and GSH regeneration. However, glutathione peroxidase (GPX), CAT, ascorbate peroxidase (APX) activities, and ascorbic acid (AsA) content decreased significantly by 69.51% (*p* < 0.05), 31.70% (*p* < 0.05), 45.22% (*p* < 0.05), and 44.04% (*p* < 0.05), respectively, suggesting impaired H_2_O_2_ detoxification systems (CAT, APX, and GPX) and disrupted AsA-GSH cycle functionality. Peroxidase (POD) activity remained unchanged (*p* > 0.05).

Exogenous Bc effectively restored antioxidant system homeostasis. Compared to the OBZ group, Bc reduced SOD activity and GSH content by 35.12% (*p* < 0.05) and 44.56% (*p*< 0.05), respectively, while enhancing CAT, POD, GPX, APX activities, and AsA content by 56.16% (*p* < 0.05), 63.35% (*p* < 0.05), 261.48% (*p* < 0.05), 83.17% (*p* < 0.05), and 70.18% (*p* < 0.05), respectively. The abnormally high GPX activity increase (261.48%) may result from Bc-mediated protection of enzymatic active sites or reduced oxidative damage via toxin adsorption. In contrast, sodium silicate (Si) exhibited differential regulation: SOD activity and GSH content decreased by 49.52% (*p* < 0.05) and 20.21% (*p* < 0.05), respectively, whereas CAT, POD, GPX, APX activities, and AsA content increased by 115.47% (*p* < 0.05), 125.07% (*p* < 0.05), 193.00% (*p* < 0.05), 38.54% (*p* < 0.05), and 48.51% (*p* < 0.05), respectively. Notably, Si outperformed Bc in activating CAT and POD (*p* < 0.05), but showed weaker efficiency in AsA recovery (48.51% vs. 70.18%, *p* < 0.05).

### 2.4. Porphyrin and Chlorophyll Metabolic Pathways

Quantitative analysis of key metabolites in chlorophyll biosynthesis revealed that OBZ treatment significantly disrupted chloroplast biogenesis in tobacco. Compared to the control (CK), glutamate (Glu) content in the OBZ group increased by 51.59% (*p* < 0.05), while δ-aminolevulinic acid (ALA), uroporphyrinogen III (Uro III), protoporphyrin IX (Proto IX), Mg-protoporphyrin IX (Mg-Proto IX), and protochlorophyllide (Pchlide) levels decreased by 28.46% (*p* < 0.05), 50.15% (*p* < 0.05), 51.96% (*p* < 0.05), 31.41% (*p* < 0.05), and 24.09% (*p* < 0.05), respectively. These results indicate that OBZ blocks chlorophyll biosynthesis by inhibiting the conversion of ALA to chlorophyll precursors.

**Figure 3:**
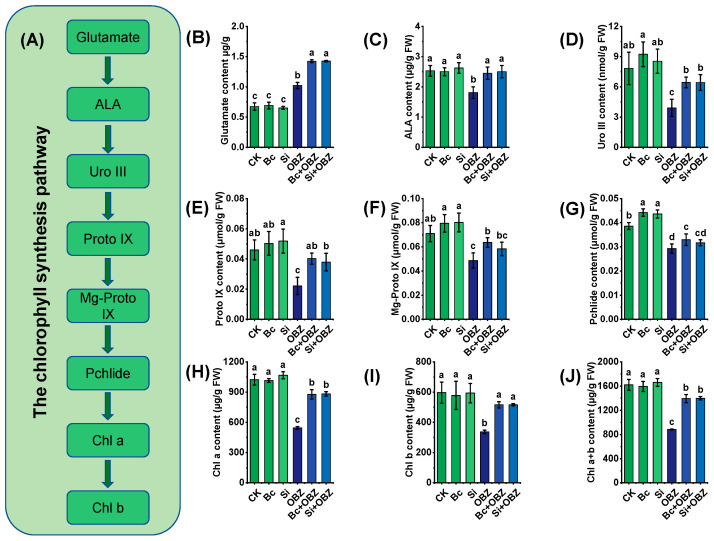
Effects of bamboo biochar (Bc) and sodium silicate (Si) on chlorophyll synthesis and chlorophyll-related metabolites in tobacco under oxybenzone (OBZ) stress. (**A**) Schematic diagram of the chlorophyll biosynthesis pathway, showing chlorophyll precursors and intermediate metabolites. (**B**–**J**) Determination of chlorophyll precursors and pigment contents: (**B**) glutamic acid, (**C**) δ-aminolevulinic acid (ALA), (**D**) uroporphyrinogen III (Uro III), (**E**) protoporphyrin IX (Proto IX), (**F**) magnesium protoporphyrin IX (Mg-Proto IX), (**G**) protochlorophyllide (Pchlide), (**H**) chlorophyll a (Chl a), (**I**) chlorophyll b (Chl b), and (**J**) total chlorophyll (Chl a + b). Data are presented as means ± standard deviation (SD, n = 3). Different lowercase letters indicate significant differences among treatments (*p* < 0.05) according to one-way ANOVA followed by Duncan’s test.

Exogenous Bc significantly restored chlorophyll precursor (Figure 3A) accumulation: Glu, ALA, Uro III, Proto IX, Mg-Proto IX, and Pchlide levels increased by 39.20% (*p* < 0.05), 35.53% (*p* < 0.05), 64.75% (*p* < 0.05), 81.59% (*p* < 0.05), 30.60% (*p* < 0.05), and 12.63% (*p* < 0.05), respectively, compared to the OBZ group. This suggests Bc may repair precursor metabolic flux by chelating toxicants or activating ALA synthase (ALAS). Sodium silicate (Si) exhibited selective regulation: Glu, ALA, Uro III, and Proto IX levels increased by 39.43% (*p* < 0.05), 38.33% (*p* < 0.05), 64.49% (*p* < 0.05), and 71.49% (*p* < 0.05), respectively, but Mg-Proto IX and Pchlide remained unchanged (*p* > 0.05). This implies that Si preferentially restores chlorophyll precursors by promoting ALA synthesis or suppressing heme branch competition.

Chlorophyll quantification further confirmed OBZ-induced inhibition: chlorophyll a (Chl a), chlorophyll b (Chl b), and total chlorophyll (Chl a + b) decreased by 46.73% (*p* < 0.05), 43.62% (*p* < 0.05), and 45.58% (*p* < 0.05), respectively, in the OBZ group. Bc restored Chl a, Chl b, and Chl a + b by 60.89% (*p* < 0.05), 53.31% (*p* < 0.05), and 57.99% (*p* < 0.05), while Si + OBZ increased them by 61.85% (*p* < 0.05), 53.17% (*p* < 0.05), and 58.54% (*p* < 0.05), respectively. The recovery effects of Bc and Si showed no significant difference (*p* > 0.05), indicating that both effectively reversed OBZ-induced chlorophyll suppression.

### 2.5. Osmotic Regulation

**Figure 4:**
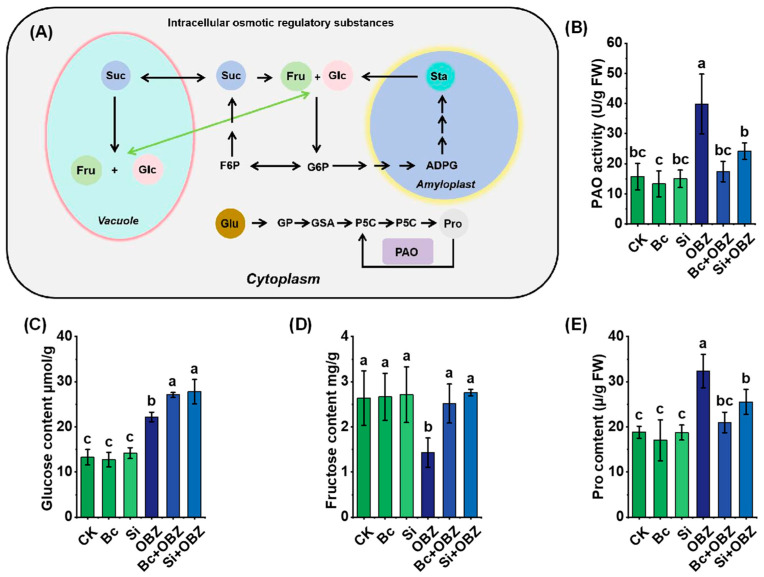
Effects of bamboo biochar (Bc) and sodium silicate (Si) on intracellular osmotic regulation and compatible solute accumulation in tobacco under oxybenzone (OBZ) stress. (**A**) Schematic illustration of osmotic adjustment pathways and key metabolic intermediates, including sugar conversion and proline biosynthesis. (**B**) Polyamine oxidase (PAO) activity. (**C**) Glucose content. (**D**) Fructose content. (**E**) Proline (Pro) content. Data are presented as means ± standard deviation (SD, n = 3). Different lowercase letters indicate significant differences among treatments (*p* < 0.05) according to one-way ANOVA followed by Duncan’s test.

Analysis of osmotic regulation-related metabolites revealed that OBZ treatment significantly altered the dynamic balance of osmolytes in tobacco (Figure 4). Compared to the control (CK), glucose, polyamine oxidase (PAO) activity, and proline content in the OBZ group increased by 67.30% (*p* < 0.05), 152.46% (*p* < 0.05), and 71.76% (*p* < 0.05), respectively, indicating that OBZ stress triggered osmolyte accumulation to counteract cellular water imbalance. Conversely, fructose content decreased by 45.67% (*p* < 0.05), suggesting reprogramming of carbon metabolism under stress.

Exogenous Bc partially reversed these metabolic disturbances: glucose and fructose levels increased by 21.91% (*p* < 0.05) and 75.86% (*p* < 0.05), respectively, compared to the OBZ group, indicating that Bc restored carbon metabolic homeostasis by promoting sugar synthesis or inhibiting degradation. Concurrently, PAO activity and proline content decreased by 56.35% (*p* < 0.05) and 35.15% (*p* < 0.05), respectively, implying that Bc alleviated osmotic stress by reducing oxidative damage or suppressing proline synthase activity.

Sodium silicate (Si) exhibited similar but distinct regulatory effects: glucose and fructose levels increased by 25.22% (*p* < 0.05) and 92.75% (*p* < 0.05), respectively, with fructose recovery significantly exceeding that of Bc (92.75% vs. 75.86%, *p* < 0.05). PAO activity and proline content decreased by 39.19% (*p* < 0.05) and 21.15% (*p* < 0.05), respectively, with smaller reductions compared to Bc (*p* < 0.05). These results suggest Si may partially substitute proline’s osmotic protective role by enhancing membrane stability or modulating polyamine metabolism.

### 2.6. Gas Exchange Parameters and Chlorophyll Fluorescence

Gas exchange parameter analysis revealed that OBZ treatment significantly inhibited photosynthetic function in tobacco. Compared to the control (CK), the net photosynthetic rate (Pn), intercellular CO_2_ concentration (Ci), transpiration rate (Tr), and stomatal conductance (Gs) in the OBZ group decreased by 54.53% (*p* < 0.05), 43.49% (*p* < 0.05), 58.44% (*p* < 0.05), and 43.97% (*p* < 0.05), respectively, indicating severe impairment of photosynthetic efficiency due to suppressed stomatal opening and carbon assimilation.

Exogenous biochar (Bc) significantly reversed this inhibition: Pn, Ci, Tr, and Gs recovered by 75.43% (*p* < 0.05), 43.34% (*p* < 0.05), 101.07% (*p* < 0.05), and 34.50% (*p* < 0.05), respectively. The most notable recovery in Tr suggests Bc alleviates stomatal limitation by adsorbing toxicants or improving water transport efficiency. Sodium silicate (Si) also exhibited strong restorative effects, increasing Pn, Ci, Tr, and Gs by 81.76% (*p* < 0.05), 41.22% (*p* < 0.05), 109.34% (*p* < 0.05), and 30.99% (*p* < 0.05), respectively. Si outperformed Bc in Tr recovery (109.34% vs. 101.07%, *p* < 0.05) but showed weaker restoration of Gs (30.99% vs. 34.50%, *p* < 0.05), implying that Si prioritizes water use optimization via transpiration-related gene regulation or enhanced cell wall rigidity.

Chlorophyll fluorescence kinetics demonstrated that OBZ severely suppressed PSII function. Compared to CK, the actual photochemical quantum yield (Y(II)), maximum photochemical efficiency (*F*v/*F*m), photochemical quenching coefficient (*q*P), non-photochemical quenching coefficient (*q*N), electron transport rate (ETR), and effective photochemical quantum yield (*F*v’/*F*m’) in the OBZ group decreased by 68.15% (*p* < 0.05), 16.24% (*p* < 0.05), 60.07% (*p* < 0.05), 10.26% (*p* < 0.05), 68.09% (*p* < 0.05), and 20.91% (*p* < 0.05), respectively. Concurrently, the regulated energy dissipation quantum yield (Y(NPQ)) increased by 42.93% (*p* < 0.05), indicating enhanced non-photochemical quenching to mitigate photodamage, while non-regulated energy dissipation (Y(NO)) remained unchanged (*p* > 0.05).

Bc partially restored PSII functionality: *F*v/*F*m, *q*P, ETR, and *F*v’/*F*m’ increased by 17.75% (*p* < 0.05), 110.08% (*p* < 0.05), 155.91% (*p* < 0.05), and 23.10% (*p* < 0.05), respectively, compared to the OBZ group, indicating thar Bc repairs light energy conversion and electron transport capacity. However, Y(II), *q*N, Y(NPQ), and Y(NO) showed no significant changes (*p* > 0.05). In contrast, Si exhibited more comprehensive PSII recovery: Y(II), *F*v/*F*m, *q*P, and ETR increased by 188.91% (*p* < 0.05), 14.29% (*p* < 0.05), 163.61% (*p* < 0.05), and 188.45% (*p* < 0.05), respectively, while Y(NPQ) decreased by 42.54% (*p* < 0.05), suggesting Si optimizes photoprotective mechanisms to reduce energy dissipation. No significant changes were observed in Y(NO), *q*N, or *F*v’/*F*m’ (*p* > 0.05).

### 2.7. Endogenous Hormones

**Figure 5:**
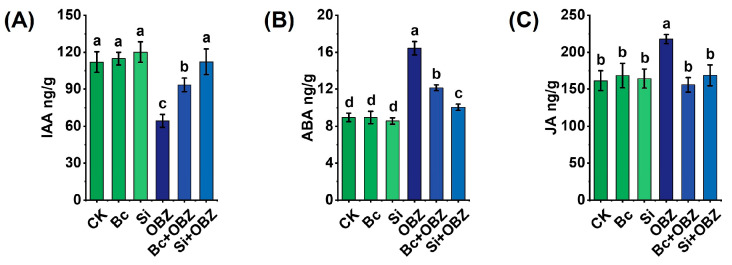
Effects of bamboo biochar (Bc) and sodium silicate (Si) on endogenous hormone levels in tobacco under oxybenzone (OBZ) stress. (**A**) Indole-3-acetic acid (IAA) content. (**B**) Abscisic acid (ABA) content. (**C**) Jasmonic acid (JA) content. Data are presented as means ± standard deviation (SD, n = 3). Different lowercase letters indicate statistically significant differences (*p* < 0.05) among treatments based on one-way ANOVA followed by Duncan’s multiple range test.

Through plant hormone content determination (Figure 5), OBZ treatment significantly altered the endogenous hormone balance in tobacco. Compared to the control group (CK), the contents of jasmonic acid (JA) and abscisic acid (ABA) in the OBZ group were significantly increased by 34.89% (*p* < 0.05) and 83.75% (*p* < 0.05), respectively, indicating that OBZ responds to oxidative damage by activating stress signaling pathways (such as JA-mediated defense responses and ABA-dependent stomatal closure); while the content of indole-3-acetic acid (IAA) was significantly decreased by 42.64% (*p* < 0.05), suggesting that OBZ may inhibit auxin synthesis or promote its degradation, thereby hindering cell elongation and organ development.

The exogenous addition of Bc effectively regulated the hormonal imbalance: compared to the OBZ group, the contents of JA and ABA decreased by 28.46% (*p* < 0.05) and 26.06% (*p* < 0.05), respectively, while the IAA content increased by 45.52% (*p* < 0.05), indicating that Bc may indirectly inhibit stress signal transduction by adsorbing OBZ toxic molecules or reducing ROS accumulation, thereby restoring auxin-driven growth processes. The addition of sodium silicate exhibited differential regulation: the contents of JA and ABA decreased by 22.53% (*p* < 0.05) and 38.85% (*p* < 0.05), respectively, and the IAA content significantly increased by 74.77% (*p* < 0.05). It is noteworthy that the Si + OBZ group exhibited a significantly stronger inhibitory effect on ABA compared to the Bc + OBZ group (38.85% vs. 26.06%, *p* < 0.05), while demonstrating a higher recovery efficiency for IAA (74.77% vs. 45.52%, *p* < 0.05). This suggests that sodium silicate may more effectively alleviate ABA-mediated stress responses by modulating ion homeostasis or enhancing cell wall integrity, while simultaneously promoting auxin synthesis or transport.

## 3. Discussion

### 3.1. ROS Accumulation and Tobacco Growth

**Figure 6:**
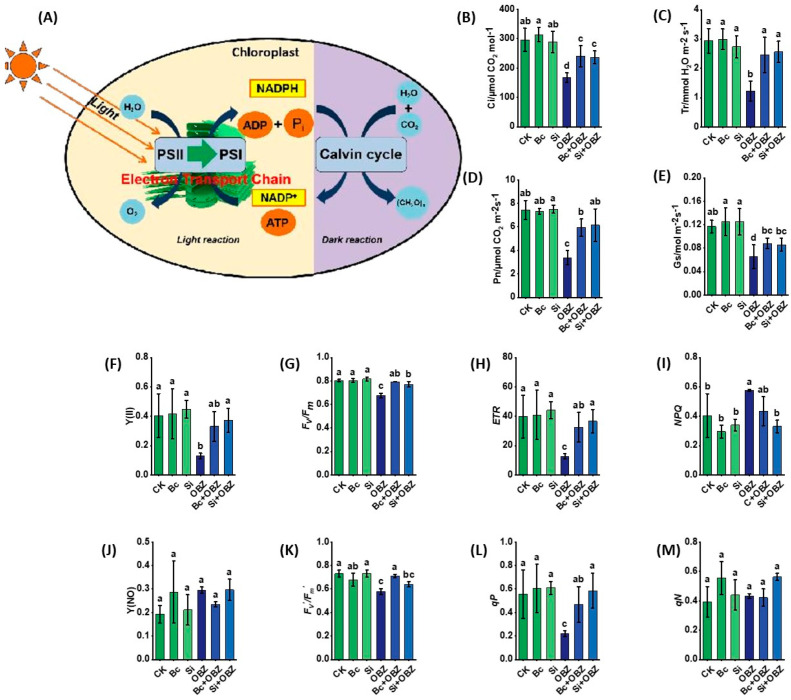
Effects of bamboo biochar (Bc) and sodium silicate (Si) on photosynthetic capacity and chloroplast function in tobacco under oxybenzone (OBZ) stress. (**A**) Schematic diagram illustrating the light reactions (PSII, PSI, and electron transport chain) and the Calvin cycle within the chloroplast. (**B**–**E**) Gas exchange parameters: (**B**) intercellular CO_2_ concentration (Ci), (**C**) transpiration rate (Tr), (**D**) net photosynthetic rate (Pn), and (**E**) stomatal conductance (Gs). (**F**–**M**) Chlorophyll fluorescence parameters: (**F**) actual photochemical quantum yield, (**G**) maximum quantum yield of PSII (*F*v/*F*m), (**H**) electron transport rate (ETR), (**I**) regulated energy dissipation quantum yield, (**J**) non-regulated energy dissipation, (**K**) effective photochemical quantum yield, (**L**) photochemical quenching coefficient, and (**M**) non-photochemical quenching coefficient. Data are presented as means ± standard deviation (SD, n = 3). Different lowercase letters indicate significant differences among treatments (*p* < 0.05) according to one-way ANOVA followed by Duncan’s test.

When plants are subjected to abiotic stress, the excessive accumulation of ROS is the core mechanism triggering oxidative damage. ROS, including O_2_^−^ and H_2_O_2_, are predominantly generated in chloroplasts, mitochondria, and peroxisomes [29,30,31]. These molecules can induce lipid peroxidation, oxidative modification of proteins, and DNA damage, ultimately leading to cellular dysfunction and, in severe cases, cell death [32,33,34]. In this study, OBZ stress significantly activated the polyamine metabolic pathway, where the accumulation of polyamines, the key metabolites in plant stress responses, aims to regulate redox homeostasis, but their oxidative degradation process catalyzed by PAO generates ROSs such as H_2_O_2_. Under OBZ treatment, PAO activity significantly increased, synergizing with the oxidative reactions induced by OBZ itself, leading to ROS accumulation far exceeding the plant’s scavenging capacity. This resulted in membrane lipid peroxidation, stomatal closure, and leaf chlorosis, manifesting as plant dwarfing and a sharp reduction in biomass (Figure 1A and Figure 6E), which is consistent both with our previous study and with the findings of Zhong et al. [35] in cucumbers. Exogenous application of Bc alleviates ROS toxicity through a dual mechanism. On the one hand, Bc reduces OBZ uptake by roots through physical adsorption, with its surface functional groups (such as carboxyl and phenolic hydroxyl groups) directly scavenging O_2_^−^ and H_2_O_2_ [36]; on the other hand, Bc improves the soil microenvironment [37], promotes nutrient absorption, and indirectly enhances the antioxidant enzyme system, thereby reducing PAO-mediated H_2_O_2_ generation. In comparison, Na_2_SiO_3_ forms a physical barrier through the silicification of cell walls, reducing the transmembrane permeation of OBZ, while optimizing the function of the mitochondrial electron transport chain to inhibit the generation of O_2_^−^ at the source and regulate the balance of polyamine metabolism [38]. The mechanisms of action of the two are significantly different: Bc is dominated by “adsorption-clearing” (a dual-action remediation mechanism where a material physically adsorbs contaminants (e.g., via van der Waals forces, π-π interactions, or pore filling) and chemically clears reactive oxygen species (ROS) through redox reactions or catalytic degradation), while Na_2_SiO_3_ focuses on “barrier-metabolic regulation” (a systemic defense strategy involving physical barrier formation (e.g., cell wall reinforcement) to block contaminant uptake, combined with reprogramming of metabolic pathways e.g., hormone signaling, antioxidant biosynthesis) to restore cellular homeostasis).

### 3.2. Changes in the Antioxidant System

The antioxidant system is the core physiological mechanism for plants to eliminate ROS, and its mechanism can be classified into two types of reaction systems: enzymatic and non-enzymatic. In the enzymatic system, SOD, CAT, APX, POD, and GPX constitute the core catalytic system; the non-enzymatic system mainly relies on antioxidant molecules such as AsA and GSH to function.

SOD, as the key enzyme for eliminating O_2_^−^, is the first line of defense of the antioxidant system against ROS [39]. This study shows that exogenous application of OBZ significantly induces an increase in SOD activity, which is speculated to be related to the stress response triggered by the excessive accumulation of O_2_^−^ in tobacco in response to OBZ stress. Notably, the addition of Bc and Na_2_SiO_3_ can significantly inhibit SOD activity, and the mechanism may involve the reduction in O_2_^−^ generation rate by both, down-regulation of SOD synthesis-related gene expression, and ultimately a decrease in the efficiency of enzyme protein synthesis. CAT and POD cooperatively regulate H_2_O_2_ levels through different catalytic pathways, where CAT specifically catalyzes the decomposition of H_2_O_2_ into H_2_O and O_2_ [40], whereas POD performs dual functions by eliminating H_2_O_2_ and oxidizing phenolic amine toxic substances. At the initial stage of stress, the activities of CAT and POD rapidly increase due to the burst of ROS; however, when the accumulation of H_2_O_2_ exceeds the critical threshold, their activities are significantly inhibited. This study found that OBZ treatment led to a decrease in CAT and POD activities, which is consistent with the mechanism of enzyme function damage caused by excessive H_2_O_2_ accumulation. Previous studies have shown that under drought, high temperature, and heavy metal stress, excessive ROS accumulation can cause multiple pathways of damage, including enzyme protein degradation, gene expression inhibition, and conformational inactivation [41,42]. This study further confirmed that Bc and Na_2_SiO_3_ treatments can effectively restore the activities of both enzymes, and the enhancing effect of Bc is more prominent [43,44,45]. Bc may enhance plant physiological functions and antioxidant capacity through improving soil physical structure (water retention, aeration), regulating pH, and promoting root development [46]. Murtaza et al. [47] and Teixeira et al. [48] studies consistently indicated that Na_2_SiO_3_ can specifically activate CAT activity, and the mechanism may be related to the regulation of the metabolic pathway mediated by phospholipase A2 (PLA2) [49].

The AsA-GSH cycle, as the core pathway for eliminating H_2_O_2_, relies on the dual-enzyme system of APX and GPX to maintain redox homeostasis [50,51]. APX uses AsA as an electron donor to catalyze the generation of monodehydroascorbic acid (MDHA), while GPX utilizes GSH to generate oxidized glutathione (GSSG), and the two enzymes work together to reduce H_2_O_2_ to H_2_O. In this study, OBZ stress led to a significant decrease in APX and GPX activities, accompanied by a decrease in AsA content and an increase in GSH levels, suggesting that the burst of ROS may exceed the regulatory threshold of the enzyme system and directly inhibit the activity of antioxidant enzymes [28]. The decrease in AsA content may be related to the regeneration disorder caused by the damage to the activities of monodehydroascorbate reductase (MDHAR) and dehydroascorbate reductase (DHAR) [52]. This study confirmed that both Bc and Na_2_SiO_3_ treatments could significantly restore the steady state of the AsA-GSH cycle. Among them, Bc mainly reduced the production of ROS by adsorbing pollutants, thereby restoring the steady state of the AsA-GSH cycle [53]; while Na_2_SiO_3_ inhibited the generation of O_2_^−^ by enhancing the cell wall structure and alleviating photoinhibition, thereby reducing the metabolic burden of the AsA-GSH cycle [54,55]. Notably, the repair efficiency of Bc was significantly better than that of Na_2_SiO_3_, which was speculated to be closely related to its stronger ROS scavenging ability and greater ability to modulate the cellular redox environment.

### 3.3. Changes in the Photosynthetic System

Chlorophyll, as a key pigment in photosynthesis, directly affects the efficiency of light energy conversion. Under OBZ stress, ROS burst inhibits chlorophyll synthesis by two primary pathways. First, synthesis of key precursor substances (ALA [56], Uro III [57], Proto IX [58], Mg-Proto IX [59,60], and Pchlide) is blocked through ROS-induced suppression of ALA synthase activity, plastid structural damage, and Mg^2+^ limitation, collectively reducing Mg-Proto IX production. Second, enhanced catabolism occurs via upregulated chlorophyllase activity accelerating pigment degradation [61], with JA-mediated senescence signals further inhibiting Pchlide accumulation [62]. At this stage, plants maintain chlorophyll dynamic balance through upregulated glutamate metabolism operating via two synergistic pathways. First, glutamate functions as both a proline synthesis precursor and osmolyte, coordinating with proline to reduce cellular osmotic potential while enhancing ROS scavenging capacity. Second, being a core nitrogen metabolism product, it supplies nitrogen sources essential for chloroplast repair [63,64,65]. However, long-term stress leads to excessive consumption of glutamate in ROS defense, exacerbating nitrogen metabolism imbalance. Bc and Na_2_SiO_3_, respectively, alleviate this process through distinct mechanisms, demonstrating their unique roles in enhancing stress tolerance. Both are capable of reducing ROS levels, minimizing glutamate consumption in the antioxidant system, and enhancing the resynthesis capability of glutamate [66]. The distinction lies in the fact that Bc promotes the uptake of soil nutrients, including magnesium ions, nitrogen, and phosphorus [67], whereas silicon enhances nitrogen assimilation efficiency [68].

PSII is highly sensitive to ROS stress [69], specifically manifested as the following: (1) damage to the donor side OEC leads to reduced oxygen evolution efficiency, with significant decreases in Y(II) and *F*v/*F*m [70]; (2) imbalance in the oxidation state ratio of the PQ pool on the acceptor side, with decreased ETR and *q*P [71]; and (3) dysregulation of heat dissipation control, with increased Y(NPQ) but failure to alleviate photoinhibition. Chloroplast ultrastructure damage and decreased LHC light-harvesting efficiency further exacerbate the barrier to light energy conversion. Bc and Na_2_SiO_3_ improve photosystem function through differentiated mechanisms. Bc enhances soil water retention capacity and mineral nutrient availability, promoting chlorophyll synthesis and regeneration of electron transport chain components (Cyt b6f, PC), increasing PSII-PSI electron flux [72,73,74,75]; while Na_2_SiO_3_ enhances cell wall mechanical strength [76], reduces chloroplast membrane lipid peroxidation (lower MDA content), and stabilizes D1 protein turnover rate [77,78]. Both can reduce Y(NO) and optimize light energy distribution efficiency.

Gs significantly decreases at the initial stage of stress [79], which is the result of the combined effect of the ABA-mediated water-saving strategy and restricted CO_2_ assimilation (lower Ci) [80]. The chain reaction manifests through two key mechanisms: Rubisco activity inhibition by insufficient NADPH supply [81], coupled with reduced Calvin cycle intermediate products, leading to decreased Pn [82]. Bc improves the rhizosphere microenvironment (adsorbing heavy metals and enhancing nutrient availability), reducing the content level of ABA, delaying stomatal closure, and thus maintaining a relatively high Gs level [83]; Na_2_SiO_3_ alleviates the damage of ROS to stomata by enhancing the activity of antioxidant enzymes, thereby maintaining stomatal opening; in addition, the reduction in ABA levels further promotes the degree of stomatal opening [84,85].

When plants are under stress, they respond to adverse environmental conditions by redistributing sugar substances. The specific mechanisms manifest through two complementary pathways: enhanced activity of starch-hydrolyzing enzymes increases glucose and fructose accumulation to maintain basal metabolism [86,87]; while heightened metabolic flux in glycolysis and the pentose phosphate pathway provides sufficient NADPH support for meeting plant metabolic demands under environmental stress [88]. Bc treatment directly increases the soluble sugar pool by enhancing Pn, and the organic carbon components it carries can serve as additional carbon sources [89,90]; Na_2_SiO_3_ reduces the loss of sugar exudation by inhibiting the increase in membrane permeability caused by MDA. This remodeling of sugar metabolism not only maintains cellular osmotic balance, but also regulates the temporal expression of stress-resistant genes through the interaction of sugar signaling (such as SnRK1) and ROS signaling [91].

### 3.4. Changes in Endogenous Hormones

Under adverse stress conditions, plants form multi-dimensional stress resistance regulation strategies by dynamically adjusting the endogenous hormone network. As a core stress signal molecule, ABA coordinates plant physiological responses through dual mechanisms of regulating stomatal movement and gene expression. On the one hand, it activates the ion channel-mediated stomatal closure in guard cells, effectively reducing water loss [92,93]; on the other hand, it induces the expression of genes related to osmoprotective substance synthesis and key enzymes in the antioxidant system, enhancing the ability to maintain cellular homeostasis [94]. This regulatory process forms a synergistic effect with the JA signal, and the two interact to jointly activate the expression of defense-related proteins, constructing a cross-resistance system against biotic and abiotic stresses [95,96]. At the same time, ABA inhibits the key enzymes of IAA synthesis and promotes its metabolic transformation, prioritizing the shutdown of growth and development programs, and strategically tilting energy resources towards stress resistance pathways [97].

Exogenous application of Bc and Na_2_SiO_3_ intervenes in the plant hormone network through differentiated action modes. Bc, with its unique physicochemical properties, reduces the absorption of stress signal molecules through adsorption, while regulating the characteristics of calcium signal oscillation and the salicylic acid synthesis pathway, indirectly inhibiting the efficiency of ABA signal transduction [98,99]. Silicon, on the other hand, reconstructs the hormone balance through dual mechanisms: in the ABA signaling pathway, the intensity of signal transduction is reduced by interfering with the synthesis of intermediate metabolites [100,101]; and it activates the biosynthesis pathway of gibberellin (GA) at the GA metabolic level, further weakening the accumulation effect of ABA by taking advantage of the GA-ABA antagonistic relationship [102]. This multi-target regulatory approach not only alleviates the metabolic consumption caused by excessive hormone system responses, but also protects the basic growth ability of plants by maintaining IAA homeostasis.

ROSs play the role of dynamic regulators in the hormone regulatory network, and their concentration threshold determines the biological effects of signal transmission [103,104]. Moderate accumulation of ROS enhances the sensitivity of ABA signals by modifying key protein residues, while excessive accumulation disrupts the IAA metabolic enzyme system and induces JA signal disorder [104,105,106]. The intervention of exogenous substances effectively regulates the balance between ROS generation and clearance, maintaining the precise transmission of hormone signals while blocking the cascade amplification effect caused by oxidative damage, ultimately achieving a dynamic balance between stress adaptation and growth and development in plants.

## 4. Materials and Methods

### 4.1. Preparation and Exposure of Experimental Materials

In this experiment, *Nicotiana tabacum* (tobacco) was used as the research subject. A substrate mixture of peat and perlite in a 3:1 ratio was prepared and sterilized at 121 °C for 30 min. Following the cooling of the substrate, tobacco seeds were sown into the seedling substrate. When the seedlings reached the six-leaf stage, they were transplanted into the same seedling substrate. In contrast to other treatments, the biochar treatment groups involved directly mixing 5% (*w*/*w*) biochar into the seedling substrate. Throughout the experiment, the environmental conditions were strictly controlled, with a temperature maintained at 25 °C, light intensity set at 300 μE, and a photoperiod of 14 h of light and 10 h of darkness.

Following transplantation, seedlings exhibiting uniform size and health were selected for simultaneous exposure and rescue experiments. Each tobacco plant received 100 mL of the corresponding treatment solution every four days, with the entire exposure period lasting 12 days. The different treatments were as follows: (1) CK, normal growth control; (2) OBZ, OBZ addition alone (100 μmol/L); (3) Bc, biochar addition alone (50 g/kg soil); (4) Si, Na_2_SiO_3_ addition alone (100 μmol/L); (5) OBZ + Bc, OBZ and biochar co-addition; and (6) OBZ + Si, OBZ and Na_2_SiO_3_ co-addition. Preparation of 10 plants per group for the above.

### 4.2. Experimental Procedures

#### 4.2.1. Phenotypic Analysis and Sample Preservation

Upon treatment completion, plants were photographed for phenotypic documentation. Three tobacco plants per treatment group were reserved for photosynthetic parameter measurements. The remaining plants were analyzed for plant height, maximum leaf length, and maximum leaf width. He fresh weight of the aerial parts was measured after these parameters were recorded. Leaves were then sampled: one leaf per treatment was punched into 8 mm diameter discs for ROS staining. The remaining leaves were divided into three portions, wrapped in aluminum foil, flash-frozen in liquid nitrogen, ground into fine powder, and stored at −80 °C for subsequent biochemical assays.

#### 4.2.2. H_2_O_2_ Content, O_2_^−^ Production Rate, and ROS Staining

H_2_O_2_ content and O_2_^−^ production rates were quantified using commercial kits (Suzhou Comin Biotechnology, Suzhou, China). Tissue samples were homogenized with extraction buffer, and measurements followed the manufacturer’s protocols.

For ROS visualization, leaf discs (8 mm diameter) were immersed in 0.1 mg/mL 3,3′-diaminobenzidine (DAB, for H_2_O_2_) or nitroblue tetrazolium (NBT, for O_2_^−^) solutions. After 24 h of dark incubation at room temperature, discs were destained in 80% ethanol at 90 °C for 20 min [107]. Images were captured using a Leica Microsystem (Leica, Wetzlar, Germany).

#### 4.2.3. Porphyrin and Chlorophyll Metabolism Assays

Glutamate content (Product Code: GLU-2-Y) was determined using a kit (Suzhou Comin Biotechnology, Suzhou, China). ALA levels were measured using a modified method from Morton (1975). Freeze-dried powder samples were mixed with pre-chilled acetate buffer (pH 4.6), vortexed for 2 min, and centrifuged to collect crude extract. The extract was mixed with acetylacetone, heated at 100 °C for 10 min for condensation, cooled, and reacted with Ehrlich’s reagent for 15 min. Absorbance at 554 nm was measured, and ALA concentration was calculated using the standard curve [108].

Proto IX, Mg-Proto IX, and Pchlide were extracted with 80% acetone under dark conditions [109]. After centrifugation, absorbance at 575, 590, and 628 nm was measured [110].

Chlorophyll content was determined by extracting samples with 95% ethanol at 4 °C for 24 h [111]. Absorbance at 665 nm and 649 nm was measured, and Chl a and Chl b concentrations were calculated using the Lambert–Beer law [112].

#### 4.2.4. Antioxidant Enzyme Activity and Metabolite Assays

Activities of CAT (Product Code: CAT-2-Y), POD (Product Code: POD-2-Y), SOD (Product Code: SOD-2-Y), APX (Product Code: APX-2-W), GPX (Product Code: GPX-2-W) and PAO (Product Code: PAO-2-G), as well as GSH (Product Code: GSH-2-W) and AsA (Product Code: ASA-2-W) levels were analyzed using kits (Suzhou Comin Biotechnology) [28,113]. For each assay, 0.1 g of powdered sample was homogenized with extraction buffer, vortexed for 3 min, and centrifuged at 4 °C. Supernatants were collected, and enzyme activities were measured following kit instructions.

#### 4.2.5. Endogenous Hormone Quantification

JA, IAA, and ABA were quantified via high-performance liquid chromatography (HPLC) according to previous publications [28,113]. Plant hormone extraction was initiated by weighing approximately 0.5 g of tissue powder into a 10 mL centrifuge tube. Samples were immersed in 5 mL of 20% (*v*/*v*) methanol and incubated at 4 °C for 16 h with intermittent shaking. Following extraction, the mixture was centrifuged at 4 °C for 10 min (10,000× *g*), and the supernatant was transferred to a graduated tube. The residual pellet was resuspended in 2 mL of 20% methanol and recentrifuged under identical conditions. Combined supernatants were adjusted to 10 mL with ddH_2_O. For purification, the crude extract was mixed with 2 mL ddH_2_O, treated with 75 μL dilute ammonia solution (1:100 *v*/*v*), and filtered through a 0.45 μm membrane. The filtrate was dried under a gentle nitrogen stream, reconstituted in 1 mL of 20% methanol, and filtered through a 0.22 μm syringe filter. A 200 μL aliquot was collected for HPLC analysis. Chromatographic separation was achieved using an Ultimate 3000RSLC system with methanol/0.075% glacial acetic acid (55:45, *v*/*v*) mobile phase at a 1.0 mL·min^−1^ flow rate. Detection wavelengths were set at 210 nm and 254 nm [114,115].

#### 4.2.6. Data Analysis and Visualization

All aforementioned parameters were measured using three biological replicates, each with three technical replicates. Data were statistically analyzed using Excel 2019 and SPSS 26. Graphs were generated using Origin 2021.

## 5. Conclusions

This study demonstrates that Bc and Na_2_SiO_3_ significantly alleviate the toxicity stress of OBZ on tobacco through differential and complementary mechanisms. Bamboo-based biochar, with its high adsorption capacity, significantly lowers ROS accumulation by directly scavenging O_2_^−^ and H_2_O_2_, thereby restoring the function of the antioxidant system and the efficiency of the AsA-GSH cycle. It also promotes chlorophyll synthesis and improves stomatal conductance, leading to a substantial recovery of photosynthetic rate. In contrast, sodium silicate enhances the mechanical barrier through cell wall silicification, reduces ROS generation, and preferentially regulates hormone balance and tissue development. It also repairs the chlorophyll precursor synthesis pathway and the function of the PSII reaction center. The targets of their actions are significantly differentiated; Bc dominates the photosynthetic recovery in the aboveground parts through physical adsorption and ROS scavenging, while Si enhances the stress adaptability of the underground parts through hormone regulation and root optimization. We envision that the combined application of Bc and Si may potentially show aboveground–underground joint repair, increasing the overall biomass and the expression of stress resistance genes in tobacco, and providing a basis for the development of composite stress resistance strategies. In the future, the ratio of the two materials should be further optimized, and their long-term impact on soil ecology should be evaluated to promote their large-scale application in agricultural pollution remediation.

## Data Availability

Data are contained within the article.
